# Feeling Informed and Safe Are Important Factors in the Psychosomatic Health of Frontline Workers in the Health Sector during the COVID-19 Pandemic in Austria

**DOI:** 10.3390/ijerph20021533

**Published:** 2023-01-14

**Authors:** Melanie Lenger, Alexander Maget, Nina Dalkner, Jorgos N. Lang, Frederike T. Fellendorf, Michaela Ratzenhofer, Elena Schönthaler, Eva Fleischmann, Armin Birner, Susanne A. Bengesser, Robert Queissner, Martina Platzer, Adelina Tmava-Berisha, Robert M. Trojak, Eva Z. Reininghaus

**Affiliations:** Clinical Department of Psychiatry and Psychotherapeutic Medicine, Medical University Graz, 8036 Graz, Austria

**Keywords:** COVID-19 pandemic, psychosomatic, worker in the health sector, stress, frontline workers

## Abstract

The global spread of the coronavirus disease (COVID-19) has created new challenges for the entire healthcare system, and those who work directly with the patients or even on the front lines with COVID-19 patients have been particularly stressed. Only a few studies are currently available investigating psychosomatic symptoms among healthcare workers, particularly frontline workers, over the entire pandemic period (2020–2022). There is also a lack of knowledge about strategies to prevent stress during and after a health crisis. Methods: An online survey was conducted at three times (April 2020, winter 2020/2021, and winter 2021/2022) during the COVID-19 pandemic in Austria. The sample included 160 healthcare workers at screening time 1, 1.361 healthcare workers at screening time 2, and 1.134 healthcare workers at screening time 3. The survey included COVID-19 work-related fears, satisfaction with the frontline work, and standardized inventories to assess psychosomatic symptoms, such as the Patient Health Questionnaire (PHQ-D). Results: Psychosomatic symptoms were more common among women compared to men, and among frontline workers compared to non-frontline workers, especially during the course of the pandemic at t2 and t3. Self-reported scores of COVID-19 work-related fears were significantly associated with psychosomatic symptoms. Furthermore, in frontline workers, there was a significant association between the feeling of being safe and well-informed and psychosomatic symptoms. Conclusion: COVID-19 work-related fears and psychosomatic symptoms have been prevalent among healthcare workers throughout the pandemic. Feeling safe and informed appears to be essential to prevent psychosomatic symptoms, leading to a recommendation for employers in the healthcare sector to focus on communication and information. As frontline workers are especially prone to psychosomatic symptoms, more stress prevention programs for them will be essential to maintain productivity and reduce sick days and fluctuations in the healthcare system.

## 1. Introduction

The severe acute respiratory syndrome coronavirus-2 (SARS-CoV-2) led to a worldwide health crisis with more than 604,465,273 cases and more than six million deaths as of 5 September 2022 according to John Hopkins University. This health crisis has been challenging for healthcare workers since the beginning of March 2020 but will affect future healthcare in general. The COVID-19 pandemic has changed the daily challenges for healthcare workers, especially for the frontline healthcare workers, but also for all the medical doctors, nurses, and therapists working with conventional somatic patients under COVID-19 safety regulations [1,2] and facing changed processes, time pressure, and lack of staff due to the pandemic [3].

The general COVID-19 safety regulations in Austria (which started in March 2020) prescribed wearing facemasks, keeping a one-meter distance from others, and restricting the number of people in one room at the same time. Shops, universities, and schools were closed, and leisure-time activities were limited. Furthermore, hand hygiene and continuous disinfection as well as protective clothing for healthcare workers were recommended. During the first lockdown in Austria from March to the beginning of May 2020, health services were limited to urgent surgeries and elective medical interventions had to be postponed. After the first lockdown, the restrictions were loosened for the general population in the summer of 2020, whereas the clinical work for the healthcare workers continued, and the numbers of COVID-19 inpatients increased continuously until September when a new lockdown started in Austria [4]. Healthcare workers were already vulnerable to stress and related psychosomatic symptoms, as well as mood disorders and anxiety disorders [5,6,7]. A study published in 2009 in Lancet showed that the pre-pandemic burnout rate for physicians and other healthcare professionals was 25% [8].

The direct contact with COVID-19 patients and the status of being key workers made social distancing even harder compared to the general population [9]. A recent study by Denning et al. (2020) [10] investigating a large sample of 2.367 healthcare workers showed that 67% of them were at high risk for burnout. Compared to studies before the COVID-19 pandemic, McKinley et al. (2020) [11] reported 31.5%, which indicates that this number increased massively due to the current health crisis. Distress at work, independent of the COVID-19 pandemic, leads to insecurity and lower quality of healthcare, and finally to decreased safety for patients [12]. Additionally, stress and fear appear more often in frontline workers and healthcare workers suffering from pre-existing somatic as well as mental disorders [13]. A meta-analysis including 93.000 healthcare workers from 21 countries reported a prevalence rate of 21.7% for depression, 21.1% for anxiety disorders, and 21.5% for post-traumatic stress disorder during the COVID-19 pandemic [14]. Female healthcare workers suffered significantly more from psychosomatic stress than their male colleagues [15]. This fact should also be considered for future gender-specific interventions to improve mental health at work for healthcare workers. Kamali et al. (2022) [16] described the feelings of healthcare workers during the pandemic of 2020–2022. Helplessness and overwhelmed feelings were demonstrated explicitly. On the other hand, protective factors like the feeling of being informed and supported are not very well understood during the COVID-19 pandemic. 

Several current publications highlight some reasons for the increased burnout rates in healthcare workers at the beginning of the crisis, such as increased challenges of learning new methods quickly, wearing protective clothing throughout the workday, feeling powerless, as well as the fear of becoming infected by COVID-19 [17,18]. Nevertheless, we have to admit that countries like Austria were not hit as hard by the COVID-19 pandemic (at least within the first lockdown) compared to several other countries, such as China, India, and Brazil, but daily challenges for frontline workers seem to have been similar around the world. Furthermore, during the crisis dealing with challenges improved but long-term consequences for mental health in healthcare workers still need to be addressed (for a review, see Shreffler et al., 2020 [19]). Psychosomatic symptoms, such as muscle tension, sleep disturbances, headache, and digestive problems, are some predominant symptoms that have been linked to work-related distress [20]. This was true before the COVID-19 pandemic but is even more relevant after 2020. 

Sakib et al. [21] found that in healthcare workers the fear of becoming infected by COVID-19 is highly associated with the development of affective disorders, such as depression. However, safety concerns and possible information providing obligations of the employer are not discussed in recent literature. In addition, studies considering the effects of the pandemic on healthcare workers in Austria are limited at the moment. Data on the impact of the COVID-19 pandemic on mental disorders in Austria for the general population is already available [22], but not especially for healthcare workers in Austria. Furthermore, we hypothesized that general healthcare workers would differ from frontline workers in Austria during the course of the COVID-19 pandemic in the extent of stress-related psychosomatic symptoms. Additionally, we hypothesized that sufficient information and the feeling of being safe are preventive factors for psychosomatic burden, depressive symptoms, and COVID-19 work-related fears. 

## 2. Materials and Methods

### 2.1. Study Design

The study was conducted at the Clinical Department of Psychiatry and Psychotherapeutic Medicine at the Medical University of Graz in Austria. This study was conducted according to the Declaration of Helsinki and was approved by the Ethics Committee of the Medical University Graz (EK-number: 32 329 ex 19/20). The ethics committee agreed to the project with the project title “Psychosocial interests on the SARS-CoV-2 pandemic on employees of healthcare in Austria”. 

During the COVID-19 pandemic (2020–2022), workers in the health sector in Austria were asked to complete an online survey via LimeSurvey (https://www.limesurvey.org/de/, accessed on 30 December 2022) at three points in time. Data collection for the first point in time started in April 2020 (t1), during the total lockdown in Austria. At this point in time, shops, schools, and universities were closed, and social distancing and home offices were recommended (except for key workers, so-called “system-relevant professions”, including a segment of healthcare workers such as doctors and nurses). Wearing a facemask was mandatory in all public buildings. At t1, 7,598 positively tested active cases were reported in Austria, while approximately 800 patients were inpatients and around 200 were patients in an intensive care unit. To follow up over the period of the pandemic, data collection for the second point in time was in the winter of 2020/2021 (t2), after the governmental restrictions had been loosened [4]. For the third point in time, the questionnaire via LimeSurvey was sent out in the winter of 2021/2022. 

The link for the online survey was sent out via work council departments and clinic management. For t1, we were only able to contact the regional hospital, but for t2 and t3, we had the chance to extend the survey link to council departments and clinic management staff of further health facilities in Austria. Data collection was anonymous. Therefore, drawing direct comparisons between t1, t2 and t3 is not possible in this study because of the divergent sample compositions. 

### 2.2. Participants 

Healthcare workers at several medical facilities in Austria (general hospitals in Austria, rehabilitation centres, the University Clinic of Graz, Austria, and foster homes around Graz, Austria) were asked to complete an online survey for three points in time. Inclusion criteria included voluntary participation (confirmation of informed consent was on the first page of the online survey), pursuing a health profession in Austria, and business e-mail availability. Additionally, more than half of the healthcare workers reported suffering at least one somatic (65.9% at t1 and 61.86% at t2) and at least one mental (76.2% at t1 and 75.94% at t2) illness. Several coping strategies of healthcare workers were reported in the online questionnaire. Twenty-two percent of the participants reported using online counselling or online therapy during the pandemic and around 25% reported taking psychopharmacological medications due to mental distress. 

### 2.3. Material and Inventories

A COVID-19-related questionnaire was created by the research team of the Clinical Department of Psychiatry and Psychotherapeutic Medicine at the Medical University of Graz, assessing COVID-19 work-related fears and COVID-19-related changes in the work routine of healthcare workers. To survey healthcare workers in the general hospital of Graz (employees of the Medical University of Graz), we used several COVID-19-related questions we formulated ourselves (please see Table 1 for an overview of the COVID-19 survey items—translated from German into English). Participants took about 20 min to complete the survey, including the informed consent at the beginning and reading general information (e.g., psychological support information) at the end of the survey. 

As a psychological inventory, the standardized Patient Health Questionnaire (PHQ-D; Gräfe et al., 2004 in the German version) was applied. The Patient Health Questionnaire (PHQ-D) screens for psychosomatic symptoms sufficiently [15,23] but also depression and anxiety [24,25]. Accordingly, those two subscales will be used for the following analyses. 

### 2.4. Statistical Analyses

Differences between frontline workers and general workers in the health sector cross-sectionally (data from April 2020, winter 2020/2021 and winter 2021/2022) were calculated with an ANCOVA (controlled for age, dependent variables: psychosomatic symptoms measured by the PHQ-D; independent variables: male vs. female, frontline vs. non-frontline workers in the health sector (WHS). The bivariate correlation analyses were calculated to assess associations between COVID-19 work-related fears and the PHQ-D score “psychosomatic symptoms” at three points in time, separately. To correct for multiple testing, the Bonferroni correction was used with an adjusted alpha level of 0.002 for *n* = 25 tests (5 scales) per point in time. 

All analyses were performed using the Statistical Package for Social Sciences (SPSS version 26.0, IBM, Armonk, NY, USA), while all data met the appropriate assumptions of multivariate normality, linearity, and homogeneity of variance. Normal distribution was confirmed using Kolmogorov-Smirnov-Tests.

## 3. Results

### 3.1. Sample Description

The specific professions in the health sector (for three points in time) are shown in Table 2. At t1, 196 participants started the online survey, and 161 participants finished the survey and have been included in the final sample. At t2, 1.759 participants opened the online survey, and 1.361 finished the survey. At t3, 1.869 participants started the questionnaire and 1.134 finished all questions. At t1, 51% were female participants, 82% were female participants at t2 and 59.9% were female participants at t3. For the distribution of the professions and the total amount of participants for all three points in time, please see Table 2. Regarding the age of the participants, please see Table 3.

Of all participants, 2.9% tested positive for COVID-19 at t1, 6.6% at t2 and 13.4% at t3. Additionally, 26.3% of the WHS reported direct contact with COVID-19 patients at t1 (41.5% of the male WHS vs. 30.6% of the female WHS), 20.3% at t2 (24.8% of the male WHS and 19.3% of the female WHS), and 49.1% at t3 (26% male WHS and 74% female WHS), and they were assigned to the frontline worker group. 

#### 3.1.1. Psychosomatic Symptoms in Workers in the Health Sector

Three ANCOVAs (t1, t2, t3) were analysed for the difference between frontline WHS and non-frontline WHS, as well as between males and females for the PHQ-D subscale psychosomatic symptoms (controlled for age). 

**Figure 1:**
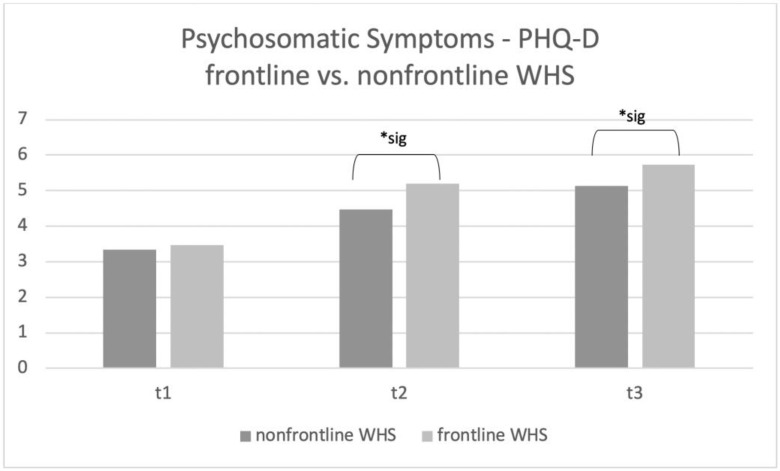
Patient Health Questionnaire (PHQ-D) subscale “psychosomatic symptoms” main effect frontline vs. non-frontline WHS. Significant results are marked with *sig.

**Figure 2:**
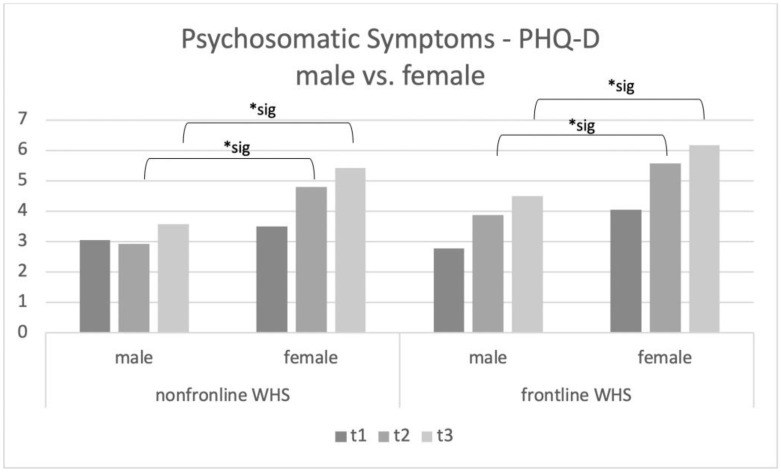
Patient Health Questionnaire (PHQ-D) subscale “psychosomatic symptoms” main effect male vs. female WHS. Significant results are marked with *sig.

In t1, there were no significant main effects for frontline vs. non-frontline WHS (F_3.541_ = 0.398; *p* = n.s.) as well as for sex (F_9.133_ = 1.026; *p* = n.s.). Frontline workers reported more psychosomatic symptoms than non-frontline WHS at t2 (F_94.95_ = 6.749; *p* = 0.009). Additionally, women showed higher numbers of psychosomatic symptoms at t2 (F_412.18_ = 29.29; *p* < 0.001). For t3, there is also a significant main effect for male vs. female (F_538.98_ = 29.17; *p* < 0.001) and frontline vs. non-frontline WHS (F_1.360_ = 0.386; *p* = n.s.). This main effect for male vs. female (F_1.360_ = 0.386; *p* = n.s.) and for frontline vs. non-frontline WHS (F_115.82_ = 0.6.270; *p* = 0.012) was significant at t3. The interactions showed no significant results at t1 (F_4.370_ = 0.491; *p* = n.s.)., t2 (F_0.884_ = 0.063; *p* = n.s.) and t3 (F_1.746_ = 0.095; *p* = 0.759). Please see Figure 1 (main effect frontline vs. non-frontline WHS) and Figure 2 (main effect male vs. female WHS) for the results of the ANCOVAs. 

#### 3.1.2. Association between COVID-19 Work-Related Fears and “Psychosomatic Symptoms”

The numbers of psychosomatic symptoms of the PHQ-D were significantly correlated to the other scores of the PHQ-D (depression and anxiety) and COVID-19 work-related fears at t1 and t3, but not at t2. Please see Table 4 for the results of the correlation analyses. Correlations between the three points in time are significantly different z = 5.1187, *p* < 0.001 *** (Fishers Z).

#### 3.1.3. Association between the Feeling of Being Informed and Safe and Psychosomatic Symptoms for the Work with COVID-19 Patients Directly 

A significant correlation between the number of psychosomatic symptoms measured by the PHQ-D at t1, t2 and t3 and the feeling of being informed and safe during work with COVID-19 patients for frontline WHS was shown at all three points in time. Please see Table 5 for the results of the correlation analyses. 

## 4. Discussion

The present study investigated mental distress, COVID-19 work-related fears, and the psychosomatic burden at three points in times during the COVID-19 pandemic in a sample of Austrian workers in the health sector. Although frontline WHS reported similar psychosomatic symptoms compared to non-frontline WHS at the beginning of the pandemic (t1). Frontline WHS showed significantly more psychosomatic symptoms compared to non-frontline WHS at t2 and t3, suggesting that continuing work directly with COVID-19 patients was, after all, massively distressing. As the governmental guidelines (including social distancing regulations) were not very strict at t2 and t3, we suggest that the burden on frontline WHS compared to general healthcare workers was higher because of the increased number of patients in the second wave of the pandemic in autumn 2020. Furthermore, female WHS reported psychosomatic symptoms more often than male WHS at t2 and t3, regardless of whether they worked on the frontline or not. Additionally, COVID-19 work-related fears were significantly associated with the number of psychosomatic symptoms. Interestingly, for the frontline WHS, the self-reported feeling of being informed and safe correlated negatively with psychosomatic symptoms. 

PHQ-D scores seem to be more conspicuous at t2, which is in line with recent literature showing long-term effects on mental well-being for healthcare workers [13,19]. Since healthcare workers are vulnerable to stress and related psychosomatic symptoms as well as affective disorders and anxiety disorders [5,6,7], the COVID-19 crisis seems to accelerate this effect. In line with that, recent studies also reported higher burnout rates for healthcare workers in 2021 than in pre-pandemic times [10]. Females reported more psychosomatic symptoms than males, which is comparable to the results of similar studies as well [15]. However, it is still unclear whether female participants are more prone to these symptoms than men or whether psychosomatic symptoms occur more often, compared to other mental illnesses [26]. A recent meta-analysis found similar findings in comparable studies investigating healthcare workers. This meta-analysis highlights the fact that female healthcare workers report 40% more often symptoms related to depression [27]. It also aligns with the facts that more female healthcare workers than male healthcare workers participate in such studies and that more women work in nursing professions, which might lead to a bias in such studies, because healthcare workers in nursing professions are even more strained than those in other professions. These effects seem to be consistent across different countries and cultures because of the high number of studies providing these results [27]. It seems to be due to the fact that women have a higher prevalence of depression and related mental symptoms in general [28]. Further investigations are needed regarding the development of psychosomatic symptoms in healthcare workers during the COVID-19 pandemic because of the already existing discrepancies in vulnerability to psychosomatic symptoms [20].

Even though Austria has not been hit very hard by the COVID-19 crisis (compared to China, India, or Brazil), the impact on mental health for frontline workers seems to be similar. New ideas from designers of work health promotions should be initiated because distress at work, independent of the COVID-19 pandemic, leads to insecurity, lower quality of healthcare, and finally, decreased safety for patients [12]. Because of the present results, we would suggest options to withdraw for key workers, such as healthcare workers, because the absence of social distancing at work might trigger psychological distress [9]. Reasons for the higher vulnerability to mental illness during the COVID-19 crisis are already known, for example increased challenges to learning new methods quickly, wearing protective clothing during the whole workday, feeling powerless, as well as the fear of becoming infected by COVID-19 [17]. Explicitly targeting these issues in supervision might reduce the daily stress levels of healthcare workers [29].

Psychosomatic symptoms, like muscle tension, sleep disturbances, headache, and digestive problems are some predominant symptoms that can be linked to work-related distress [20]. This was true before the COVID-19 pandemic but is even more relevant after the year 2020. The PHQ-D screens somatoform disorders sufficiently [30], but in recent literature the PHQ-D has been used to determine depression and anxiety in healthcare workers [24,25], whereas psychosomatic symptoms should be accounted for equally [15]. 

Factors that reduce vulnerability to stress-related disorders at work for healthcare professionals are, for example, yoga and physical exercise (for a review see [31]) or mindfulness training [32]. The positive effect of functional coping mechanisms to deal with daily challenges in the COVID-19 pandemic is also discussed in recent literature [33,34,35]. However, clear guidelines to prevent stress during the COVID-19 pandemic for healthcare workers to prevent psychophysiological burdens are still rare. 

The present study highlights the mental impact of the COVID-19 crisis on frontline workers in Austria; similar to the study by Pieh et al. [22], who highlighted the mental impact of the COVID-19 crisis on the general population in Austria. 

Furthermore, it focuses on the importance of information from the employer. Information might be one step towards preserving mental health in healthcare workers, as was highlighted by Sakib et al. [21], who reported the association between the fear of becoming infected by COVID-19 and the development of affective disorders, such as depression. In the present study, we found a relationship between the subjective feeling of being safe at the workplace and psychosomatic symptoms, which are very often anxiety-related. The relationship between psychological burdens like psychosomatic symptoms, depression, and anxiety are also highlighted in a recent meta-analysis [36]. The present results are in line with these findings and provide information about the relationship between these parameters. 

Murthy addresses healthcare workers’ well-being in general and, in case of a new worldwide health crisis, in a very recent publication [37]. This publication highlights physical protection, the importance of strengthening the healthcare infrastructure in general and the improvement of public opinion to reduce discrimination based on race, gender, or disabilities. Additionally, it focuses on lacking access to mental health care for healthcare workers that does not lead to stigmatization. These present results, as well as results from comparable studies, support this suggestion and future projects to improve the mental health of healthcare workers. 

### Limitations

Since participation in this survey was voluntary and anonymous, it is not possible to compare the whole datasets from three points in time, even though some subjects participated three times. In addition, the total number of participants differed between t1 and t2, and the number of nurses is much higher at t2 than at t1, which should be considered in the interpretation of the results. The lack of significant results at t1 might be due to the low number of participants. Effects would have been recognizable with a larger sample size, similar to t2 and t3. Since the survey was sent out via comparable email lists, we are able to theoretically compare the main assumptions in the interpretation. 

In addition, we do not know what the level of psychosomatic symptoms was in frontline and non-frontline workers before the pandemic, so we can only speculate that the clear difference between t2 and t3 between frontline and non-frontline workers is due to the high stress caused by working with the COVID-19 patients. 

The questionnaires and self-reported scales give no insight into objective procedures in healthcare facilities. However, since the mental impact of the COVID-19 pandemic is always measured subjectively, the present investigation is comparable. Standardized questionnaires with clinical implications, such as the PHQ-D, give a good overview of the actual situation for healthcare workers in Austria. 

Female WHS are predominant in the current sample at both points in time. This is comparable to recent studies using online questionnaires for the general population [38], as well as for samples in the healthcare sector [15] that report having about 60% female participants. This might be due to the motivation to answer surveys like this. 

The scale “Feeling Informed and Safe” consists only of general questions about the subjective feeling of being informed by the employer and the government and one interoceptive question about the general feeling of safety. Since there is no questionnaire available about job security and safety at the workplace during the COVID-19 pandemic, new standardized questionnaires are needed. Furthermore, other parameters to measure the quality of work and the satisfaction during the COVID-19 pandemic need to be included in further surveys. This research field needs more investigation. The present results provide first steps in that direction. 

## 5. Conclusions

Frontline WHS in Austria are prone to psychosomatic symptoms, and related differences in general healthcare workers were observed during the course of 2020–2022. Information and provision of infrastructure for feeling safe are important factors for the mental health of healthcare workers, especially during the COVID-19 pandemic. Psychosomatic symptoms are related to the feeling of being informed and safe. Furthermore, COVID-19 work-related fears were more common in general healthcare workers than in frontline workers at the beginning, while psychosomatic symptoms and mood disturbances seem to appear more often in frontline workers due to the ongoing crisis. To protect healthcare workers from emotional disturbances and stress-related psychiatric disorders, clear information, access to safety infrastructure, and possibilities for dealing with psychosomatic symptoms (e.g., offers from workplace health promotion programs, such as resilience training focused on healthcare staff) are essential. 

## Figures and Tables

**Table 1:** COVID-19-related survey.

Topic	Questions	Answer Options
COVID-19 work-related fears	During the COVID-19 pandemic, I am anxious about… …my job. …the health of my relatives. …my own health. …the commercial crisis. …nothing.	Yes/No
Feeling save and informed (only for frontline workers in the health sector	I feel sufficiently informed for my work with COVID-19 patients and my own safety by my employer. I feel sufficiently informed for my work with COVID-19 patients and my own safety by the government. During the work with COVID-19 patients, I feel safe.	I agree/I disagree

Note. Questions in the LimeSurvey Questionnaire for all three points in time.

**Table 2:** Professions of the Healthcare Workers in both samples.

Healthcare Profession	t1	t2	t3
Medical Doctors	70	150	160
Medical Doctors in Training	28	45	60
Psychologists and Therapists	6	185	86
Medical Technical Assistants	2	123	165
Scientific Staff	74	33	45
Nurses or Nursing Assistants	0	777	290
Facility Management	0	5	4
Administrative Staff	0	174	181

Note. Number of participants for that profession group. Therapists include physiotherapists and occupational therapists.

**Table 3:** Age distribution at two time-points.

Age in Years	t1	t2	t3
18–30	12.5	20.4	491
31–40	27.6	19.8	421
41–50	15.9	19.8	414
51–60	18.5	16.5	350
61–70	3.9	1.9	35
71–80	0.4	0.0	1

Note. Numbers describe percentages.

**Table 4:** Correlations between the psychosomatic score and COVID-19 work-related fears.

**t1**					
		PHQ-D psychosomatic symptoms	PHQ-D depression	PHQ-D anxiety	COVID-19 work-related fears
PHQ-D psychosomatic symptoms	r	1	0.577 **	0.056	0.392 **
	*p*		0.000	0.808	0.000
**t2**					
		PHQ-D psychosomatic symptoms	PHQ-D depression	PHQ-D anxiety	COVID-19 work- related fears
PHQ-D psychosomatic symptoms	r	1	0.589 **	−0.107	0.004
	*p*		0.000	0.221	0.968
**t3**					
		PHQ-D psychosomatic symptoms	PHQ-D depression	PHQ-D anxiety	COVID-19 work- related fears
PHQ-D psychosomatic symptoms	r	1	0.582 **	−0.187 *	0.178 **
	*p*		0.000	0.021	0.000

Note. ** *p* < 0.001; * *p* < 0.005.

**Table 5:** Correlations between the psychosomatic score and the score “Feeling informed and safe”.

Feeling Informed and Safe		PHQ-D Psychosomatic Symptoms	COVID-19 Work-Related Fears
t1	*r*	−0.371 **	−0.171
	*p*	0.007	0.226
t2	*r*	−0.193 **	−0.122
	*p*	0.002	0.691
t3	*r*	−0.191 **	−0.088 *
	*p*	0.000	0.027

Note. ** *p* < 0.001; * *p* < 0.005.

## Data Availability

Not applicable.
